# Probe-based confocal laser endomicroscopy for real-time diagnosis of multifocal gastric mucosa-associated lymphoid tissue lymphoma

**DOI:** 10.1055/a-2885-8127

**Published:** 2026-06-19

**Authors:** Yulu Zheng, Xinjian Wan, Zhixia Dong

**Affiliations:** 1 Digestive Endoscopic CenterShanghai Jiaotong University School of Medicine Affiliated Sixth People’s HospitalShanghaiChina

**Keywords:** Endoscopy lower GI tract, Diagnosis and imaging (inc chromoendoscopy, NBI, iSCAN, FICE, CLE...), Endoscopy upper GI tract, Precancerous conditions and cancerous lesions (displasia and cancer) stomach, Endoscopic resection (ESD, EMRc, ...)

The diagnosis of gastric mucosa-associated lymphoid tissue (MALT) lymphoma remains challenging. Highly variable endoscopic findings and the risk of sampling errors in multifocal lesions result in a low positive identification rate on pathological examination, posing great difficulty for early detection. 1​ ​2​ More recently, probe-based confocal laser endomicroscopy (pCLE) has emerged as a novel tool enabling real-time in vivo histological assessment, offering a potential solution. In the present case, we demonstrate for the first time the application of pCLE for the real-time visualization of neoplastic lymphoid infiltration in multifocal gastric MALT lymphoma ( Video 1 ).

**Video 1** In vivo real-time histological imaging of gastric MALT lymphoma by pCLE shows the loss of a glandular architecture and dense lymphoid cell infiltration, facilitating the accurate and targeted diagnosis of multifocal gastric MALT lymphoma.


A 50-year-old woman presented with epigastric discomfort and underwent upper gastrointestinal endoscopy. Conventional endoscopy revealed multiple whitish lesions in the greater and lesser curvature of the gastric body, ranging from 1.5 to 3.5 cm in size ( Fig. 1a–b ). Magnifying narrow-band imaging (ME-NBI) showed the absence of normal glandular structures and abnormal dilated microvessels ( Fig. 1c–f ). pCLE revealed the complete loss of the glandular architecture with dense infiltration of numerous small dark cells and increased vascular density, corresponding to the histopathological findings of diffuse infiltration of centrocyte-like cells and lymphoepithelial lesions ( Fig. 2 ). Immunohistochemistry revealed positivity for CD79a and Bcl-2 and negativity for CyclinD1, CD10, Bcl-6, MUM1, and CD23. These findings supported the diagnosis of gastric MALT lymphoma ( Fig. 3 ).

**Fig. 1:**
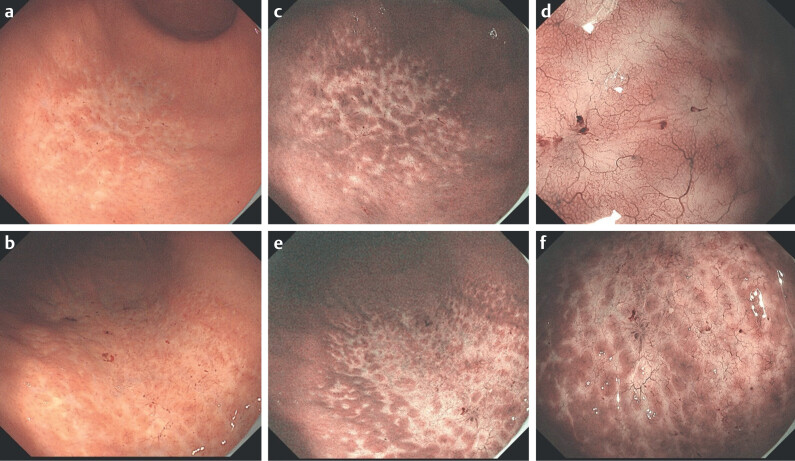
Endoscopic features of multifocal gastric MALT lymphoma. ( **a** and **b** ) Conventional endoscopy shows multiple whitish mucosal lesions measuring 1.5–3.5 cm in the greater and lesser curvature of the gastric body. ( **c** – **f** ) ME-NBI reveals the loss of the normal glandular architecture and abnormally dilated microvessels.

**Fig. 2:**
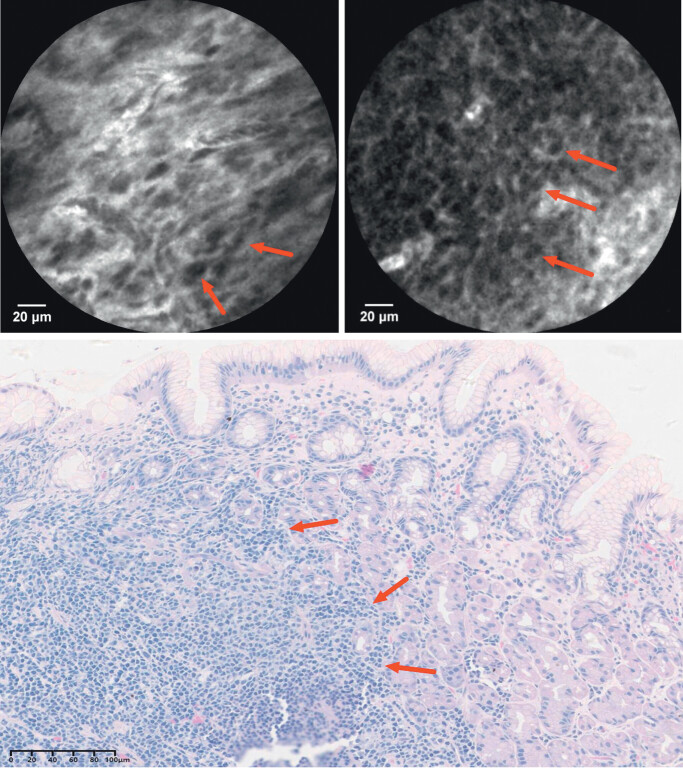
pCLE imaging and corresponding histopathological features of gastric MALT lymphoma. pCLE demonstrates the complete loss of the normal glandular architecture, dense infiltration of numerous small dark cells, and increased vascular density (red arrow). These findings correlate with histopathological evidence of diffuse centrocyte-like cell infiltration and lymphoepithelial lesions (red arrow).

**Fig. 3:**
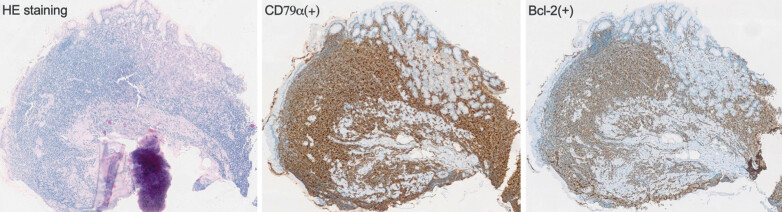
Immunohistochemical confirmation of gastric MALT lymphoma. Immunohistochemistry revealed positivity for CD79a and Bcl-2.

In the diagnosis of gastric MALT lymphoma, pCLE elevates biopsy from random sampling to precision navigation by providing real-time, cellular-level pathological images. Rather than replacing histopathology as the gold standard, it serves as a powerful adjunct that enables endoscopists to directly visualize target lesions, thereby improving the accuracy of targeted biopsy, reducing missed diagnoses, and minimizing unnecessary tissue sampling. The combination of ME-NBI and pCLE may further enhance diagnostic accuracy for subtle and multifocal gastric MALT lymphoma, offering a significant advantage over conventional endoscopy alone by reducing diagnostic delays and enabling earlier treatment.

Endoscopy_UCTN_Code_CCL_1AB_2AD_3AB

## Notice

This article was changed according to the erratum on July 27, 2026.

## Erratum

In the above-mentioned article the Author order was changed. This was corrected in the online version on 27th of July 2026.
