# Correction: Probe-based confocal laser endomicroscopy for real-time diagnosis of multifocal gastric mucosa-associated lymphoid tissue lymphoma

**DOI:** 10.1055/a-2917-1402

**Published:** 2026-08-11

**Authors:** Yulu Zheng, Xinjian Wan, Zhixia Dong

**Affiliations:** 1 Digestive Endoscopic CenterShanghai Jiaotong University School of Medicine Affiliated Sixth People’s HospitalShanghaiChina



In the above-mentioned article the Author order was changed. This was corrected in the online version on 27th of July 2026.

